# Inhibition of eIF2*α* Dephosphorylation Protects Hepatocytes from Apoptosis by Alleviating ER Stress in Acute Liver Injury

**DOI:** 10.1155/2020/2626090

**Published:** 2020-06-05

**Authors:** Yong-Jing Tang, Huan Chen, Yu Yi, Gui-Mei Chen, Fang-Wan Yang, Ying Li, Ren-Dong Tian, Wen-Ge Huang, Qi-Jiao Cheng, Yi-Huai He

**Affiliations:** Department of Infectious Diseases, The Affiliated Hospital of Zunyi Medical University, Zunyi, 563003 Guizhou, China

## Abstract

**Objectives:**

Protein kinase R-like ER kinase (PERK)/eukaryotic initiation factor 2 alpha (eIF2*α*) is an important factor along the main pathways for endoplasmic reticulum (ER) stress-mediated apoptosis. In this study, we investigated the effects of eIF2*α* phosphorylation on hepatocyte apoptosis and the ER stress mechanisms in acute liver injury.

**Methods:**

eIF2*α* phosphorylation and apoptosis under ER stress were monitored and measured in male BALB/c mice with acute liver injury and human hepatocyte line LO2 cells.

**Results:**

Carbon tetrachloride (CCl_4_) administration triggered ER stress and hepatocyte apoptosis, as well as eIF2*α* phosphorylation in mice. Inhibition of eIF2*α* dephosphorylation, as the pretreatment with 4-phenylbutyric acid (chemical chaperone, ER stress inhibitor), mitigated CCl_4_-induced intrahepatic ER stress, apoptosis, and liver injury. In an ER stress model of LO2 cells induced by thapsigargin (disrupting ER calcium balance), inhibition of eIF2*α* dephosphorylation reduced ER stress and apoptosis, while PERK knockdown reduced eIF2*α* phosphorylation and exacerbated ER stress and apoptosis.

**Conclusions:**

eIF2*α* phosphorylation is one of the mechanisms employed by ER stress for restoring cellular homeostasis. Inhibition of eIF2*α* dephosphorylation mitigates hepatocyte apoptosis by alleviating ER stress in acute liver injuries.

## 1. Introduction

Liver injury can be initiated by a variety of causes, including infection with hepatitis viruses, alcohol, drugs, metabolic abnormalities, autoimmunity, ischemia, and hypoxia [1]. However, hepatocyte injury remains the most common pathophysiological basis of various liver diseases and the main cause of liver dysfunction [2]. Apoptosis, as it relates to a form of hepatocyte injury, can be triggered by intra- or extracellular signaling. Endoplasmic reticulum (ER) stress is one of the intracellular signaling pathways for mediation of apoptosis. ER stress is initiated when unfolded/misfolded proteins accumulate in the ER and bind to glucose-regulated protein 78 (GRP78) [3]. This particular binding event leads to phosphorylation of protein kinase R-like ER kinase (PERK) and inositol-requiring enzyme 1 alpha (IRE1*α*) and cleavage of the activating transcription factor 6 (ATF6) [4, 5]. Phosphorylated PERK can thereby also phosphorylate the alpha subunit of eukaryotic initiation factor 2 (eIF2*α*). Current theory suggests that phosphorylated eIF2*α* represses protein synthesis and reduces protein load in the ER [6]. On the other hand, the phosphorylated eIF2*α* selectively induces the response of activating transcription factor 4 (ATF4) [7, 8], which regulates the expression of GRP78, growth arrest and DNA damage 34 (GADD34), and C/EBP homologous protein (CHOP). Research further suggests that GADD34 can interact with protein phosphatase 1 (PP1), thereby dephosphorylating eIF2*α* and effectively forming a negative feedback loop to restore protein synthesis [9]. ER stress results in proteolytic cleavage of ATF6, generating a 50 kD active fragment [10], whereby ATF6 activation leads to an increased transcription of a network of genes, including GRP78 and X-box binding protein 1 (XBP1). Koh et al. discovered that spliced XBP1 (XBP1s) is converted from a nonspliced isoform by IRE1*α* endonuclease, facilitating the expression of a number of unfolded protein response (UPR) responsive genes [11, 12], similar to the types of UPRs found in ER stress environments.

**Figure 1:**
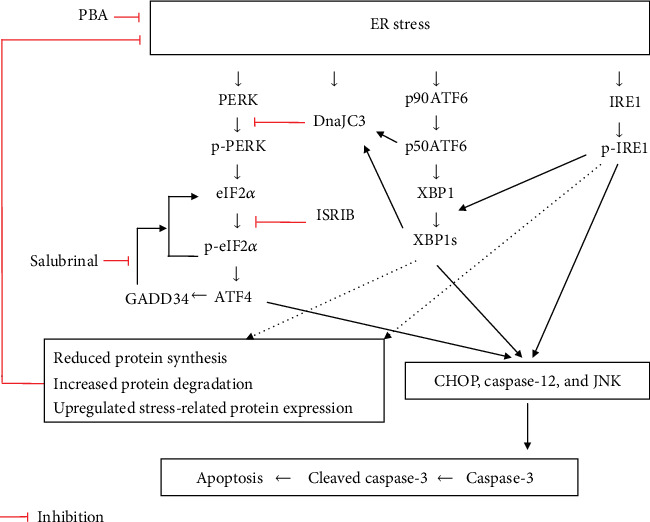
The impact of ER stress on apoptosis. PERK/eIF2*α* is an important factor in the main pathways for ER stress-mediated apoptosis. eIF2*α* integrates multiple signals and involves both prosurvival and proapoptotic pathways of ER stress.

While research suggests a multitude of naturally occurring ER stress regulators, studies continue to demonstrate the efficacy of ER stress regulation *via* chemical treatment. 4-Phenylbutyric acid (PBA, a chemical chaperone) alleviates ER stress in a variety of cell types [13, 14]. Salubrinal, a treatment alternative method, selectively suppresses eIF2*α* dephosphorylation by inhibiting PP1 activity, sustaining the phosphorylated eIF2*α* status, while ISRIB inhibits the eIF2*α* phosphorylation [15–17]. In addition, DnaJC3 is an ER stress-regulated chaperone and can inhibit eIF2*α* kinases including PERK, protein kinase R (PKR), general control nonderepressible 2 (GCN2), and heme-regulated inhibitor (HRI) [18, 19]. Taken together, PERK, ATF6, and IRE1*α* can impede protein synthesis, upregulate an ER response protein, activate ER-related degradation, and promote cell survival [20]. If ER homeostasis is disturbed, ER stress will trigger proapoptotic signaling, such as CHOP, c-Jun N-terminal kinase (JNK), and caspase-12 [21, 22]. Caspase-3 responds to both intra- and extracellular signals and is subject to cleavage in an effort to initiate apoptosis [23, 24]. The impact of ER stress on apoptosis is shown in Figure 1.

ER stress inevitably occurs in the pathogenesis of various liver diseases [25, 26]. The PERK/eIF2*α* relationship provides a key component for the resulting ER stress-mediated apoptosis [27]. This study utilized a carbon tetrachloride- (CCl_4_, through conversion into reactive trichloromethyl to injure the liver) induced acute liver injury mouse model and a thapsigargin- (TG, through disruption of the ER calcium balance) induced ER stress model in cultured hepatocytes to determine the effect of inhibited eIF2*α* dephosphorylation on hepatocyte apoptosis and investigated in detail the molecular mechanism.

## 2. Materials and Methods

### 2.1. Animals and Induction of Liver Injury

Male BALB/c mice (18 ± 2 g) were supplied by the Animal Center of Zunyi Medical University (Guizhou, China) and housed in a specific pathogen-free facility where room temperatures varied between 20 and 24°C. Mice were acclimated for one week prior to the start of experimental procedures, where they were then monitored for health and behavior every 12 h. Before the experimental procedure was initiated, investigators and technicians were educated by ethics experts on experimental animal welfare and animal use ethics. All mouse studies were carried out in accordance with the guidelines of China Animal Care and Research. The animal study protocol was approved by the Animal Care and Use Committee of the Affiliated Hospital of Zunyi Medical University (Guizhou Province, China).

To induce acute liver injury, mice were randomly grouped and injected intraperitoneally with 10 mL/kg body weight of olive oil alone (control) or a mixture of CCl_4_ (25%) and olive oil (75%) (acute liver injury model group, *n* = 10). In regulating ER stress chemically, mice were pretreated with salubrinal (1 mg/kg body weight, vehicle: dimethyl sulfoxide (DMSO); Sigma), ISRIB (2.5 mg/kg body weight, vehicle: phosphate buffer solution (PBS); Sigma), or PBA (150 mg/kg body weight, vehicle: PBS; Sigma) for 2 h and then administered CCl_4_ for 24 h, resulting in salubrinal+CCl_4_, ISRIB+CCl_4_, and PBA+CCl_4_ groups (*n* = 10). For those control groups, mice were injected with the same dose of salubrinal, ISRIB, or PBA before the injection of olive oil, resulting in salubrinal, ISRIB, or PBA sham groups. Transduction of the mice occurred, whereby recombinant adeno-associated virus vector serotype 8 (AAV8) expressing DnaJC3 (rAAV8-DnaJC3, NM-008929, GeneChem, Shanghai, China) or control AAV8 (2 × 10^10^ viral genome copies in 100 *μ*L PBS per mouse) was injected through the tail vein. CCl_4_ was administrated 4 weeks after transduction, producing the DnaJC3+CCl_4_ and AAV8+CCl_4_ control groups (*n* = 8), respectively.

### 2.2. Humane Endpoints

At the conclusion of each experiment, mice were sacrificed unless they died prematurely. One to three mice were placed in a 4000 mL euthanasia box. The box chamber air was then replaced with 100% carbon dioxide at a turnover rate of 30% of the chamber volume per minute. Mice were anesthetized under these conditions for up to 3 minutes. After losing consciousness and presenting an inability to respond to stimuli, blood circulation was maintained while tissue and blood were harvested. The entire anesthetization procedure was performed within 10 minutes.

### 2.3. Cell Culture and Induction of ER Stress

LO2 cells (an isolated human normal hepatocyte line) were purchased from the Cell Bank of the Type Culture Collection (Chinese Academy of Sciences, Shanghai). LO2 cells were cultured in RPMI 1640 medium supplemented with 10% fetal bovine serum (Gibco, USA) and 1% penicillin/streptomycin. To induce ER stress, 2 × 10^5^ LO2 cells/well grown in a 6-well plate were treated with TG (0.5 *μ*mol/L, 4 mL/well; Sigma, St. Louis, MO, USA) or DMSO for 12, 24, and 48 h. To investigate the effect of eIF2*α* phosphorylation on apoptosis under ER stress, LO2 cells were pretreated with salubrinal (20 *μ*mol/L, Sigma) for 2 h before the inclusion of DMSO (control) or TG (0.5 *μ*mol/L, Sigma).

### 2.4. PERK Short Hairpin RNA Transfection

A total of 1.2 × 10^6^ LO2 cells/well were seeded in a 6-well plate and cultured for 24 h. Once cells reached 80% confluence, they were transfected with plasmids encoding short hairpin RNA (shRNA) targeting the human PERK gene (5′-GCACTTTAGATGGGAGAATTGCGAACAATTCTCCCATCTAAAGTGC-3′; control shRNA: 5′-AAACGTGACACGTTCGGAGAA CGAATTCTCCGAACGTGTCACGTTT-3′) (purchased from GeneChem, Shanghai, China) in the presence of Lipofectamine 3000, for 5 h. The transfected cells were incubated for an additional 43 h with 2 mL/well normal growth medium after the removal of transfection mix. Finally, ER stress was induced by the addition of 0.5 *μ*mol/L TG for another 24 h. This experiment resulted in two treatment groups: PERK shRNA+TG and control shRNA+TG groups.

### 2.5. Western Blot Analysis

Fifty milligrams of autopsied liver tissues was homogenized in 5 mL of immunoprecipitation assay lysis buffer (R0010, Solarbio, Beijing, China). Individual liver or LO2 cell lysates (40 *μ*g/lane) were separated in 10-12% sodium dodecyl sulfate polyacrylamide gel electrophoresis (SDS-PAGE), and the gel was transferred to polyvinylidene fluoride membranes (Millipore, 0.45 *μ*m, Billerica, MA, USA). The membranes were blocked with 5% fat-free dry milk in Tris-buffered saline with Tween 20 (TBST) and probed with mouse monoclonal antibodies against ATF6 (MA1-25358, Thermo Fisher Scientific, USA), *β*-actin (sc-58673, sc: Santa Cruz Biotechnology), CHOP (ab11419, ab: abcam), and PERK (sc-377400) and rabbit monoclonal antibodies against ATF4 (11815, Cell Signaling Technology), cleaved caspase-3 (9664, Cell Signaling Technology), DnaJC3 (MA5-14820, Thermo Fisher Scientific, USA), phosphorylated eIF2*α* (p-eIF2*α*, 3398, Cell Signaling Technology), phosphorylated PERK (p-PERK, MA5-15033, Thermo Fisher Scientific, USA), and XBP1s (83418, mouse: 55 kD, human: 60 kD, Cell Signaling Technology). The bound antibodies were detected with horseradish peroxidase- (HRP-) conjugated anti-mouse or anti-rabbit IgG and visualized by enhanced chemiluminescent reaction. The relative levels of target protein compared to the control were determined *via* densitometry, using Quantity One software (Bio-Rad, Hercules, CA, USA). In addition, a single membrane can be probed multiple times after striping earlier bound antibodies. Briefly, the visualized membranes were immersed in stripping buffer for 10-60 minutes to remove the bound antibodies and then primed for incubation with a new set of antibodies against a different protein.

### 2.6. Histology and Immunohistochemistry

Liver tissues were fixed in 10% formalin and embedded in paraffin. Sections (5 *μ*m thickness) were stained with hematoxylin and eosin (HE). In addition, the tissue sections were subjected to immunohistochemistry using monoclonal antibodies against cleaved caspase-3 (9664, Cell Signaling Technology) and CHOP (ab11419). The stained sections were viewed under a light microscope (Olympus CX31). The liver histology was independently scored by two experienced pathologists using the Histology Activity Index- (HAI-) Knodell score. Image-Pro Plus 6.0 was used for quantitative analysis of histology and immunohistochemistry [28].

### 2.7. Serum Alanine Aminotransferase Level

Blood samples were collected from each mouse upon euthanasia. Serum alanine aminotransferase (ALT) levels were determined using a rate method (the Beckman Coulter autoanalyzer, AU5800, USA) [29].

### 2.8. Cell Viability Assay

The MTS assay was used to determine the extent of cell viability with the [3-(4,5-dimethylthiazol-2-yl)-5-(3-carboxymethoxyphenyl)-2-(4-sulfophenyl)-2H-tetrazolium] CellTiter 96® AQueous One Solution Cell Proliferation Assay Kit (Promega Corporation, Fitchburg, WI, USA). In brief, 20 *μ*L LO2 cell culture medium was replaced with MTS solution, and cells and solution were incubated at 37°C for 3 h. Next, cell viability was measured colorimetrically at 490 nm in a microplate reader (Model 680, Bio-Rad). The cell viability was normalized as a percentage of the control as previously described [30].

### 2.9. Cell Apoptosis Analysis

Apoptosis was determined with the TransDetect Annexin V-FITC/PI Cell Apoptosis Detection Kit (TransGen Biotech, Beijing, China) using flow cytometry. The apoptotic index was calculated as the percentage of annexin V-positive cells divided by the total number of cells in the gated region as previously outlined [31].

### 2.10. Statistical Analysis

The normally distributed data were expressed as mean ± standard deviation (SD). Differences between groups were statistically analyzed using the one-way analysis of variance (ANOVA) with Tukey's post hoc analysis. The one-sample Kolmogorov-Smirnov test was used to analyze the normality of distribution for continuous variables. A *P* value of <0.05 was considered statistically significant [32].

## 3. Results

### 3.1. CCl_4_ Administration Induces Intrahepatic ER Stress and Apoptosis in Mice

**Figure 2:**
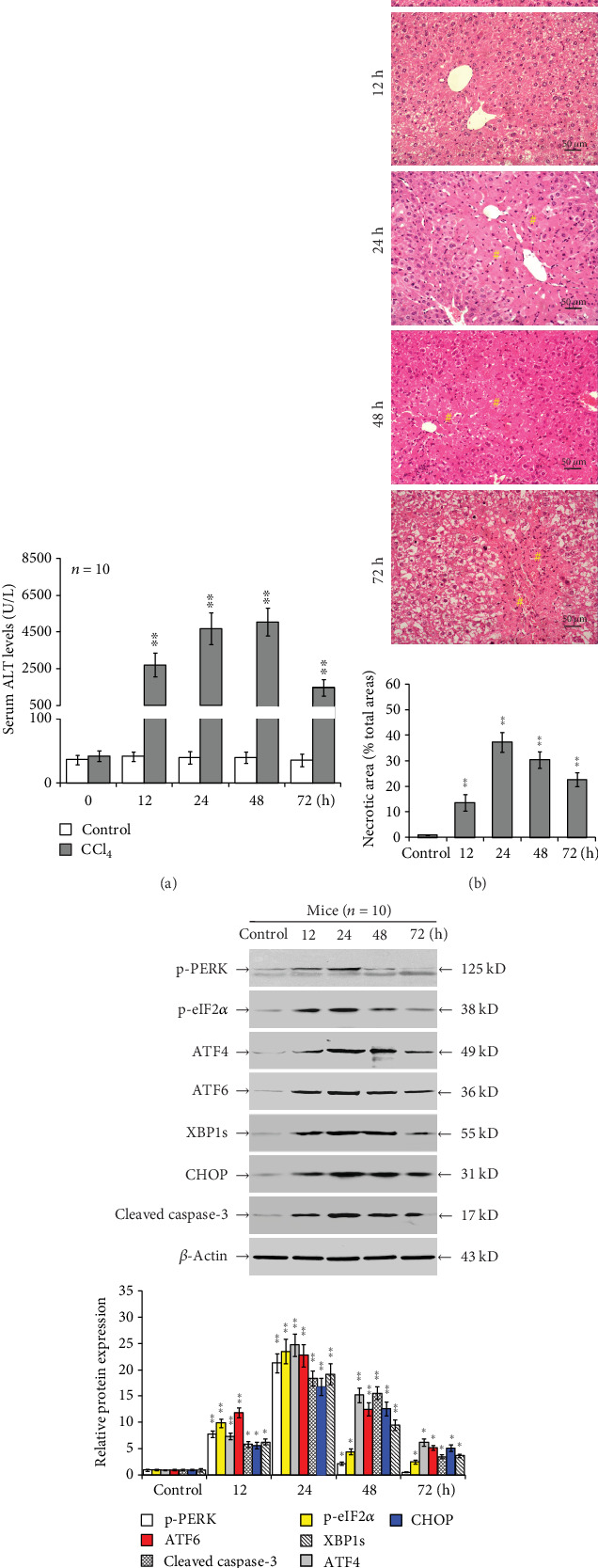
CCl_4_ administration induces intrahepatic ER stress and apoptosis in mice. Male BALB/c mice were randomly grouped and injected with olive oil (control) or CCl_4_ (*n* = 10, total mice = 100). (a) Serum ALT levels determined by the rate method. (b) Liver histology (magnification ×100). Hash sign indicates the necrotic area. (c) Relative protein levels of ER stress and hepatocyte apoptosis in mouse livers at the indicated time points postinjection were determined by Western blot. Representative blots and histology from four independent experiments are shown. Histograms represent mean ± SD of four independent experiments. ^∗^*P* < 0.05, ^∗∗^*P* < 0.01 versus the control group.

Serum ALT levels were elevated (*P* < 0.01; Figure 2(a)), hepatocyte necrosis occurred (*P* < 0.01; Figure 2(b)), and upregulated intrahepatic p-PERK, p-eIF2*α*, ATF4, ATF6 (a 36 kD band corresponding to the cleaved form of ATF6), XBP1s, CHOP, and cleaved caspase-3 protein expression peaked at 24 h post-CCl_4_ injection (*P* < 0.05; Figure 2(c)).

### 3.2. Inhibition of eIF2*α* Dephosphorylation Mitigates the CCl_4_-Induced Apoptosis and Liver Injury in Mice

**Figure 3:**
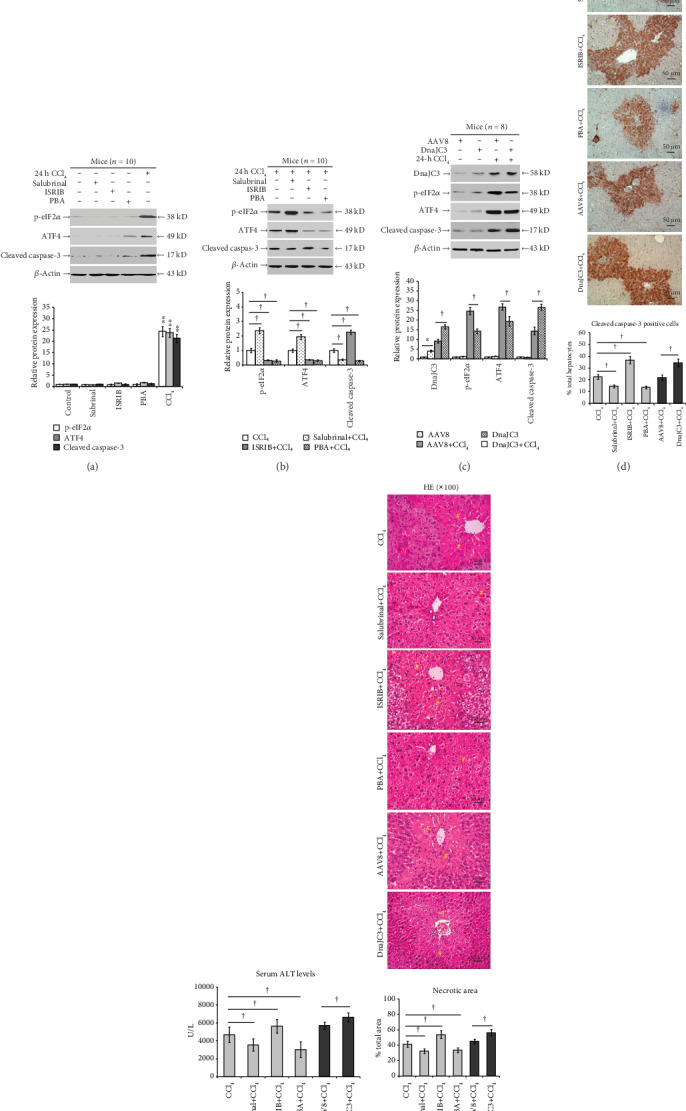
Inhibition of eIF2*α* dephosphorylation mitigates CCl_4_-induced apoptosis and liver injury in mice. Male BALB/c mice were pretreated with a vehicle (DMSO+PBS, control), salubrinal, ISRIB, or PBA for 2 h or with the recombinant AAV8 that expressed DnaJC3 for 4 weeks and then injected with olive oil or CCl_4_ for another 24 h. (a) Levels of intrahepatic p-eIF2*α*, ATF4, and cleaved caspase-3 in control, salubrinal, ISRIB, PBA, and CCl_4_ groups. (b) Salubrinal, ISRIB, PBA, or (c) the overexpressed DnaJC3 altered the levels of DnaJC3, p-eIF2*α*, AFT4, and cleaved caspase-3 in the liver. (d) Immunohistochemistry staining of cleaved caspase-3 expression in the liver (magnification ×100). (e) Kinetic serum ALT levels among different groups of mice. (f) Liver histology (magnification ×100). Hash sign indicates the necrotic area. Representative blots, immunohistochemistry, and histology from four independent experiments are shown. Histograms represent mean ± SD of four independent experiments (*n* = 8-10, total mice = 122). ^∗^*P* < 0.05, ^∗∗^*P* < 0.01 versus the control group. ^†^*P* < 0.05 versus the CCl_4_ or AAV8+CCl_4_ group.

As shown in Figure 3(a), salubrinal, ISRIB, or PBA pretreatment alone did not significantly change intrahepatic p-eIF2*α*, ATF4, and cleaved caspase-3 protein expression in mice without liver injury, suggesting that the phosphorylation of eIF2*α* is contingent upon stimulation as identified in liver injury. Once injury was induced in our mouse model, the salubrinal pretreatment significantly increased the intrahepatic p-eIF2*α* and ATF4 protein expression (*P* < 0.05; Figure 3(b)). ISRIB and DnaJC3 overexpression or PBA pretreatment significantly downregulated the CCl_4_-induced p-eIF2*α* and ATF4 protein expression. However, pretreatment with salubrinal or PBA significantly reduced the cleaved caspase-3 protein levels, which were augmented by ISRIB or DnaJC3 overexpression pretreatment in CCl_4_-induced mice (*P* < 0.05; Figure 3(c)). Similar patterns were obtained in liver sections by immunohistochemistry (*P* < 0.05; Figure 3(d)).

Serum ALT levels were significantly reduced with the salubrinal or PBA pretreatment and significantly elevated by the pretreatment with ISRIB or DnaJC3 overexpression post-CCl_4_ injection (*P* < 0.05; Figure 3(e)). Histologically, though the necroinflammation in mice pretreated with salubrinal or PBA developed, the extent and severity of hepatic necrosis were significantly reduced (*P* < 0.05; Figure 3(f)). In contrast, the hepatic necrosis was significantly increased in ISRIB or DnaJC3 overexpression-pretreated mice. These data indicate that inhibition of eIF2*α* dephosphorylation may mitigate CCl_4_-induced apoptosis and liver injury by alleviating ER stress.

### 3.3. Inhibition of eIF2*α* Dephosphorylation Mitigates ER Stress in Response to CCl_4_ Injury

**Figure 4:**
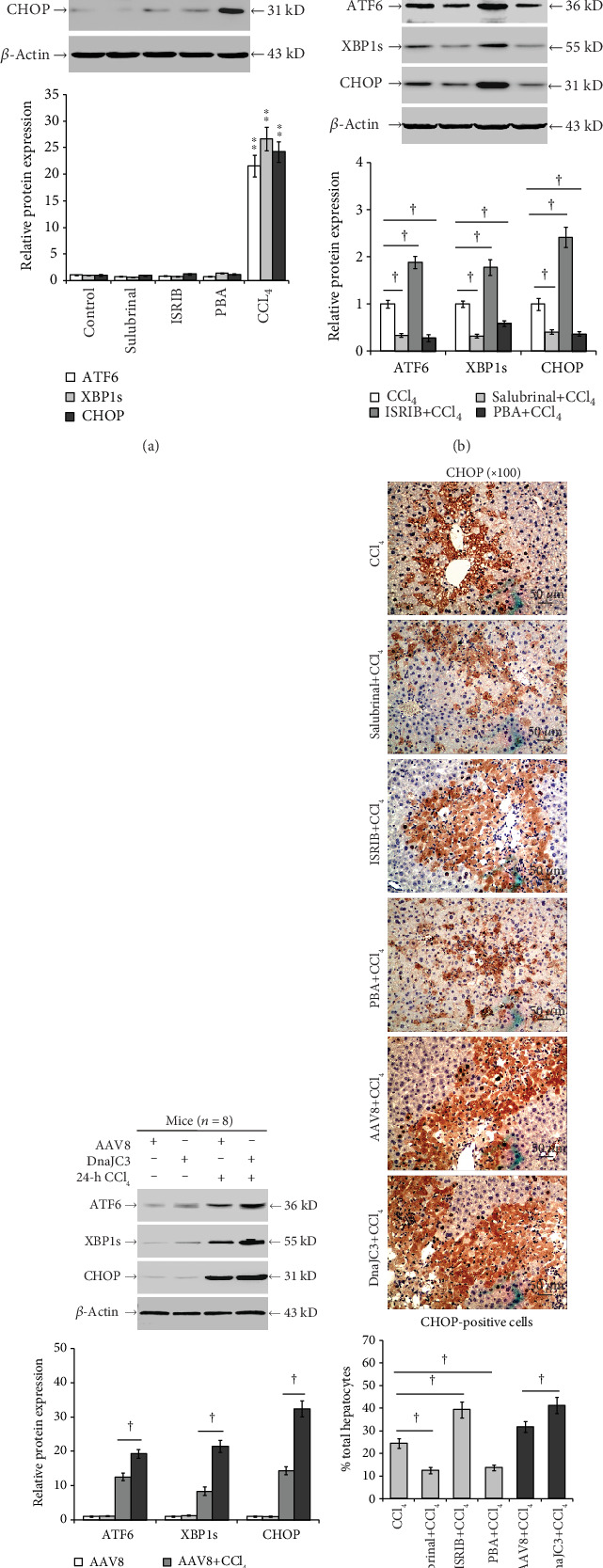
Inhibition of eIF2*α* dephosphorylation mitigates ER stress in response to CCl_4_ injury. (a) Levels of intrahepatic ATF6, XBP1s, and CHOP in control, salubrinal, ISRIB, PBA, and CCl_4_ groups. (b) Salubrinal, ISRIB, or (c) the overexpressed DnaJC3 altered the expression of intrahepatic ATF6, XBP1s, and CHOP which was determined by Western blot. (d) Immunohistochemistry staining of CHOP expression in the liver (magnification ×100). Representative blots and immunohistochemistry from four independent experiments are shown. Histograms represent mean ± SD of four independent experiments (*n* = 8-10). ^∗∗^*P* < 0.01 versus the control group. ^†^*P* < 0.05, ^††^*P* < 0.01 versus the CCl_4_ or AAV8+CCl_4_ group.

As shown in Figures 4(a) and 4(b), the CCl_4_-induced ATF6, XBP1s, and CHOP expression in the liver was significantly reduced by the salubrinal or PBA pretreatment and significantly increased by ISRIB or DnaJC3 overexpression pretreatment (*P* < 0.05; Figure 4(c)). The immunohistochemistry staining resulted in similar patterns as seen with the intrahepatic CHOP expression, as detected by Western blot (*P* < 0.05; Figure 4(d)).

### 3.4. Inhibition of eIF2*α* Dephosphorylation Reduces TG-Induced ER Stress and Apoptosis in LO2 Cells

**Figure 5:**
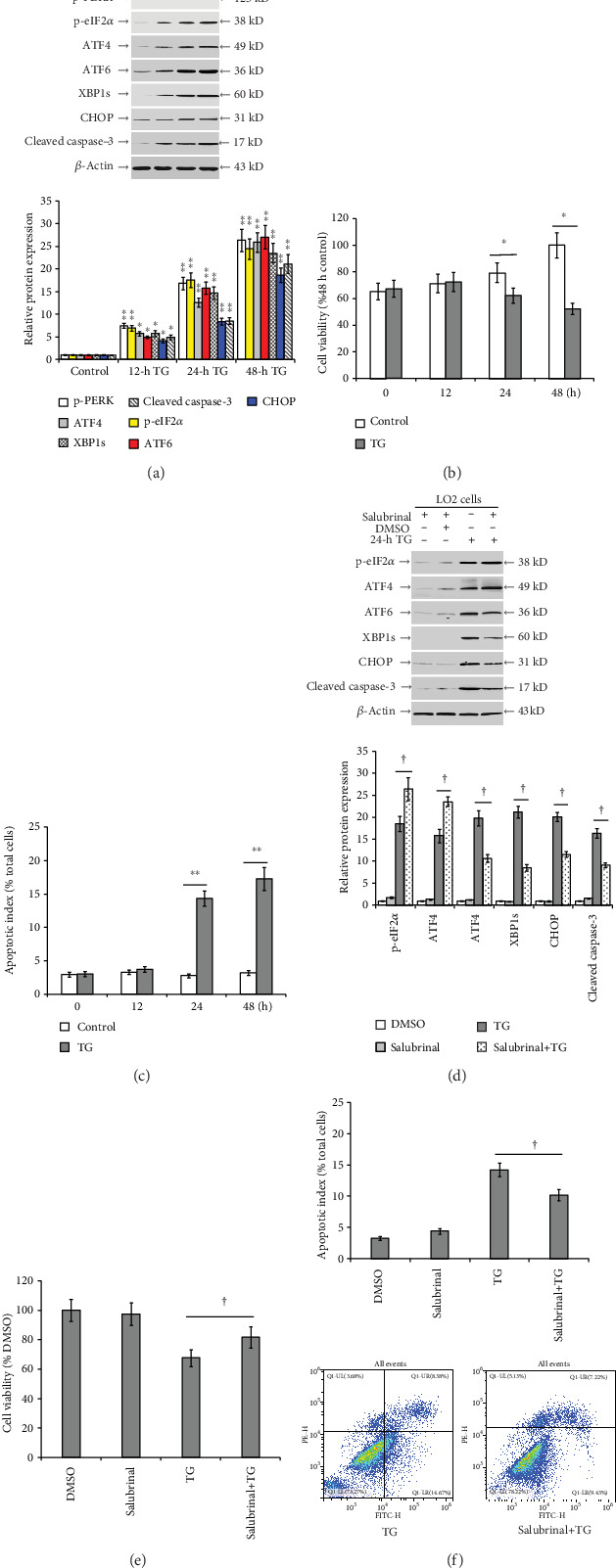
Inhibition of eIF2*α* dephosphorylation reduces TG-induced ER stress and apoptosis in LO2 cells. (a) p-PERK, p-eIF2*α*, ATF4, ATF6, XBP1s, CHOP, and cleaved caspase-3 protein expression at 12, 24, and 48 h post-DMSO (control) or post-TG (0.5 *μ*mol/L) incubation in LO2 cells. (b) Viability of LO2 cells determined by MTS. (c) Bar chart representing the apoptotic index in LO2 cells determined by flow cytometry. (d) p-eIF2*α*, ATF4, ATF6, XBP1s, CHOP, and cleaved caspase-3 protein expression in control (DMSO pretreatment for 2 h and DMSO incubation for 24 h), salubrinal (salubrinal pretreatment for 2 h and DMSO incubation 24 h), TG (DMSO pretreatment for 2 h and TG incubation 24 h), and salubrinal+TG (salubrinal pretreatment for 2 h and TG incubation 24 h) groups. (e) Viability of LO2 cells determined by MTS. (f) Apoptotic index in LO2 cells determined by flow cytometry. Representative blots from four independent experiments are shown. Histograms represent mean ± SD of four independent experiments. ^∗^*P* < 0.05, ^∗∗^*P* < 0.01 versus the control group. ^†^*P* < 0.05 versus the TG group.

TG treatment of LO2 cells significantly increased p-PERK, p-eIF2*α*, ATF4, ATF6, XBP1s, CHOP, and cleaved caspase-3 protein expression (*P* < 0.05; Figure 5(a)), reduced cell viability (*P* < 0.05; Figure 5(b)), and elevated apoptotic index (*P* < 0.05; Figure 5(c)). No significant increase was measured in p-eIF2*α*, ATF4, ATF6, XBP1s, CHOP, and cleaved caspase-3 protein expression, and no impact on cell viability and apoptotic index was detected in salubrinal-pretreated LO2 cells without ER stress. However, pretreatment with salubrinal significantly increased the TG-induced p-eIF2*α* and ATF4 protein expression, reduced ATF6, XBP1s, CHOP, and cleaved caspase-3 protein expression (*P* < 0.05; Figure 5(d)), and partially restored cell viability (*P* < 0.05; Figure 5(e)) as well as reducing apoptotic index (*P* < 0.05; Figure 5(f)).

### 3.5. PERK Knockdown Reduces TG-Induced eIF2*α* Phosphorylation and Aggravates ER Stress and Apoptosis in LO2 Cells

**Figure 6:**
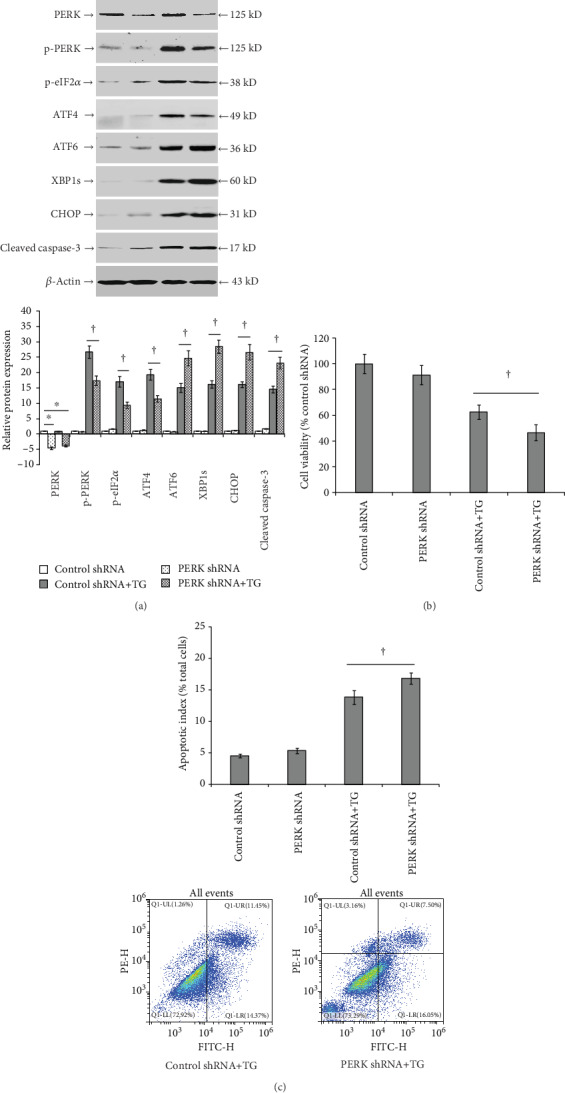
PERK knockdown aggravates TG-induced ER stress and apoptosis in LO2 cells. LO2 cells were pretreated with PERK shRNA or control RNA for 48 h and then incubated with or without TG (0.5 *μ*mol/L) for another 24 h. (a) Detected protein levels of PERK, p-PERK, p-eIF2*α*, ATF4, ATF6, XBP1s, CHOP, and cleaved caspase-3 in control shRNA (control shRNA pretreatment and DMSO incubation), PERK shRNA (PERK shRNA pretreatment and DMSO incubation), control shRNA+TG (control shRNA pretreatment and TG incubation), and PERK shRNA+TG (PERK shRNA pretreatment and TG incubation) groups by Western blot. (b) Bar chart showing the cell viability among the different experimental groups as determined by the MTS assay. (c) Cell apoptotic index determined by flow cytometry. Representative blots from four independent experiments are shown. Histograms showing the mean ± SD of four independent experiments. ^∗^*P* < 0.05 versus the control shRNA group. ^†^*P* < 0.05 versus the control shRNA+TG group.

Compared to control-treated cells, the expression of PERK was significantly lowered following PERK shRNA pretreatment in TG-untreated LO2 cells (*P* < 0.05; Figure 6(a)). The decreased PERK expression significantly suppressed TG-induced p-PERK, p-eIF2*α*, and ATF4 protein expression and increased ATF6, XBP1s, CHOP, and cleaved caspase-3 protein levels. Furthermore, compared to the TG group, the cell viability was significantly reduced (*P* < 0.05; Figure 6(b)), and the apoptotic index was significantly increased in the PERK shRNA+TG group (*P* < 0.05; Figure 6(c)).

## 4. Discussion

In the present study, we investigated the impacts of eIF2*α* phosphorylation on hepatocyte apoptosis and evaluated the therapeutic implication in acute liver injury. We investigated hepatocyte apoptosis that presented an increased phosphorylation of eIF2*α* as a direct result of CCl_4_ administration in mice and TG incubation in LO2 cells. Inhibition of eIF2*α* dephosphorylation mitigated ER stress and hepatocyte apoptosis in mice and LO2 cells, as well as alleviating liver injury in mice. These results confirm that eIF2*α* phosphorylation is one of the cytoprotective mechanisms for ER stress. To our knowledge, this is the first study suggesting that inhibition of eIF2*α* dephosphorylation mitigates hepatocyte apoptosis in acute liver injury.

CCl_4_ can be converted into reactive trichloromethyl by cytochrome P450 2E1, which induces hepatic lipid peroxidation, oxidative stress, ER stress, and inflammation, leading to hepatocyte degeneration, apoptosis, and other injuries [33, 34]. ER stress is a defensive response to a host of stimulants, but it can evolve to encompass cell injury [35]. PERK/eIF2*α* is one of the main pathways for ER stress-mediated apoptosis [36]. However, recent studies have shown that increasing eIF2*α* phosphorylation protected injured cells [37]. In this study, ER stress, as well as eIF2*α* phosphorylation, and hepatocyte apoptosis were induced through CCl_4_ administration in mice. Inhibition of eIF2*α* dephosphorylation, as a direct result of a decrease in ER stress by PBA, significantly mitigated hepatocyte apoptosis and liver injury. Conversely, the pretreatment with ISRIB or DnaJC3 overexpression inhibited CCl_4_-induced eIF2*α* phosphorylation and increased hepatocyte apoptosis and liver injuries. Our data further supports the previous claims that the elevation of eIF2*α* phosphorylation moderates hepatocyte apoptosis [38, 39]. However, PBA pretreatment, which reduced eIF2*α* phosphorylation, also lessened the degree of CCl_4_-induced hepatocyte apoptosis. PBA, a chemical chaperone, can relieve ER stress through enhancing a prosurvival response and is shown to protect various cells [40, 41]. Previous studies confirm that the effective rescuer of ER stress can mitigate liver injury [42, 43]. In addition, CCl_4_ administration induced eIF2*α* dephosphorylation as well as apoptosis in mice. Therefore, eIF2*α* phosphorylation does not directly regulate hepatocyte apoptosis in acute liver injury. Instead, the inhibition of eIF2*α* dephosphorylation may mitigate CCl_4_-induced hepatocyte apoptosis through alleviating the cellular ER stress.

PERK, ATF6, and IRE1*α* pathways mediate ER stress and have been shown to trigger both prosurvival and proapoptosis pathways [44, 45]. Although eIF2*α* phosphorylation may mediate ER stress-related apoptosis, as suggested by some studies, more recent studies demonstrated that increased eIF2*α* phosphorylation protected injured cells. In this study, we found that the PBA pretreatment decreased the CCl_4_-induced eIF2*α* phosphorylation and the expression of ATF6, XBP1s, and CHOP and exerted a form of hepatic protection, suggesting that PBA rescued the ER stress and reduced the need for the eIF2*α* phosphorylation-mediated protection. Salubrinal pretreatment inhibited the eIF2*α* dephosphorylation and mitigated ER stress *in vivo*. Adversely, ISRIB or DnaJC3 overexpression pretreatment reduced the intrahepatic eIF2*α* phosphorylation but aggravated ER stress. By inhibiting its dephosphorylation, eIF2*α* has elevated phosphorylation levels and may therefore have enhanced abilities to regulate ER stress, ultimately restoring homeostasis. Furthermore, salubrinal pretreatment inhibited TG-induced eIF2*α* dephosphorylation and reduced ER stress and apoptosis, while a low level of PERK phosphorylation reduced TG-induced eIF2*α* phosphorylation and aggravated ER stress and apoptosis in LO2 cells. These results support that eIF2*α* phosphorylation coupled with ATF6 and IRE1/XBP1 signals to regulate ER stress, and these three pathways together determine the initiation of ER stress-related apoptosis. Inhibition of eIF2*α* dephosphorylation mitigates apoptosis through decreasing ER stress.

## 5. Conclusion

Inhibition of eIF2*α* dephosphorylation mitigated intrahepatic ER stress and hepatocyte apoptosis in acute liver injury. Our findings provide new insights into the impacts of hepatic eIF2*α* phosphorylation on liver injury.

## Data Availability

The datasets generated and analyzed during the current study are available from the corresponding author on reasonable request.
